# The ubiquitous mitochondrial protein unfoldase CLPX regulates erythroid heme synthesis by control of iron utilization and heme synthesis enzyme activation and turnover

**DOI:** 10.1016/j.jbc.2021.100972

**Published:** 2021-07-16

**Authors:** Catherine M. Rondelli, Mark Perfetto, Aidan Danoff, Hector Bergonia, Samantha Gillis, Leah O'Neill, Laurie Jackson, Gael Nicolas, Herve Puy, Richard West, John D. Phillips, Yvette Y. Yien

**Affiliations:** 1 Department of Biological Sciences, University of Delaware, Newark, Delaware, USA; 2 Pittsburgh Heart, Lung and Blood Vascular Medicine Institute and Department of Medicine, University of Pittsburgh, Pittsburgh, Pennsylvania, USA; 3 Division of Hematology, University of Utah School of Medicine, Salt Lake City, Utah, USA; 4 Centre de Recherche sur l'inflammation, Université Paris Diderot, Site Bichat, Sorbonne Paris Cité, Paris, France; 5 Centre Français des Porphyries, Hôpital Louis Mourier, APHP, Colombes, France; 6 Delaware Biotechnology Institute, University of Delaware, Newark, Delaware, USA

**Keywords:** heme, iron, ATP-dependent protease, mitochondria, ferrochelatase, protein degradation, 5-aminolevulinate synthase, protoporphyrinogen IX oxidase, porphyria, AAA+, ATPases associated with various cellular activities, ALAS, aminolevulinate synthase, CLPP, caseinolytic protease proteolytic subunit, CLPX, caseinolytic mitochondrial matrix peptidase chaperone subunit X, EPP, erythropoietic protoporphyria, FECH, ferrochelatase, MEL, mouse erythroleukemia, PPIX, protoporphyrin IX, PPOX, protoporphyrinogen IX oxidase

## Abstract

Heme plays a critical role in catalyzing life-essential redox reactions in all cells, and its synthesis must be tightly balanced with cellular requirements. Heme synthesis in eukaryotes is tightly regulated by the mitochondrial AAA+ unfoldase CLPX (caseinolytic mitochondrial matrix peptidase chaperone subunit X), which promotes heme synthesis by activation of δ-aminolevulinate synthase (ALAS/Hem1) in yeast and regulates turnover of ALAS1 in human cells. However, the specific mechanisms by which CLPX regulates heme synthesis are unclear. In this study, we interrogated the mechanisms by which CLPX regulates heme synthesis in erythroid cells. Quantitation of enzyme activity and protein degradation showed that ALAS2 stability and activity were both increased in the absence of CLPX, suggesting that CLPX primarily regulates ALAS2 by control of its turnover, rather than its activation. However, we also showed that CLPX is required for PPOX (protoporphyrinogen IX oxidase) activity and maintenance of FECH (ferrochelatase) levels, which are the terminal enzymes in heme synthesis, likely accounting for the heme deficiency and porphyrin accumulation observed in *Clpx*^*−/−*^ cells. Lastly, CLPX is required for iron utilization for hemoglobin synthesis during erythroid differentiation. Collectively, our data show that the role of CLPX in yeast ALAS/Hem1 activation is not conserved in vertebrates as vertebrates rely on CLPX to regulate ALAS turnover as well as PPOX and FECH activity. Our studies reveal that CLPX mutations may cause anemia and porphyria *via* dysregulation of ALAS, FECH, and PPOX activities, as well as of iron metabolism.

Heme is a prosthetic group comprising a central iron chelated by a tetrapyrrole ring. It is critical for many life-essential redox processes, such as detoxification, oxygen transport, circadian rhythm, and control of transcription and translation (1, 2, 3). Most of the body's heme is synthesized in differentiating red cells, whose main function is to transport oxygen *via* hemoglobin (4). Proteins that regulate mitochondrial metabolism play essential roles in heme regulation (5, 6, 7, 8). However, the interactions between mitochondrial homeostasis and heme synthesis, and the extent to which these interactions are tissue-specific, are poorly understood.

Heme synthesis is tightly regulated by mitochondrial CLPX, a member of the ubiquitous AAA+ (*ATPases associated with various cellular activities*) protein unfoldase family (9). CLPX is a ring-shaped homo-hexamer and is best understood for its function in a proteasome-like enzyme complex with the peptidase CLPP (caseinolytic protease proteolytic subunit). The complex of CLPX and CLPP together forms the CLPXP ATP-dependent protease. The CLPXP protease is best understood to facilitate degradation of misfolded mitochondrial proteins. CLPX recognizes specific sequences in protein substrates and unfolds protein tertiary structures through its central pore, presenting the unfolded polypeptide chain to the CLPP proteolytic chamber (10, 11). Although CLPXP functions as a protease, detailed studies on yeast and vertebrate cells indicate that CLPX has functions that are distinct from its role in CLPXP. While *Clpp*^*−/−*^ mice survive to adulthood (12, 13, 14), *Clpx*^*−/−*^ mouse embryos die before gastrulation (15). *Saccharomyces cerevisiae* express *Clpx* (*Mcx1*), but lack a *Clpp* homolog, suggesting that CLPX regulates protein unfolding independent of proteolysis (9).

Insights from model organisms show that CLPX can regulate heme synthesis by mediating activation (9) and degradation (16) of the ALAS enzymes, which catalyze the committed step of heme synthesis. Yeast ALAS (Hem1) requires CLPX (Mcx1) for its full activation; as yeast lack a CLPP ortholog, CLPXP does not regulate Hem1 stability in yeast. *Clpxa*-deficient zebrafish were anemic, suggesting that CLPX was required for heme synthesis (9). These studies predicted that CLPX deficiency will cause accumulation of inactive ALAS protein and heme deficiency. However, erythroid cells expressing an ATPase inactive CLPX Gly298Asp mutant protein (ATPase activity being necessary for CLPX fucntion (9)) had *increased* ALAS protein and ALAS activity. The elevated ALAS activity caused erythropoietic protoporphyria (EPP) resulting from accumulation of protoporphyrin IX (PPIX) (17) (Fig. 1*A*). These results suggested that regulation of heme synthesis by CLPX was not conserved across species, necessitating additional work to understand the role of CLPX in erythroid heme regulation and in hematologic diseases.

**Figure 1:**
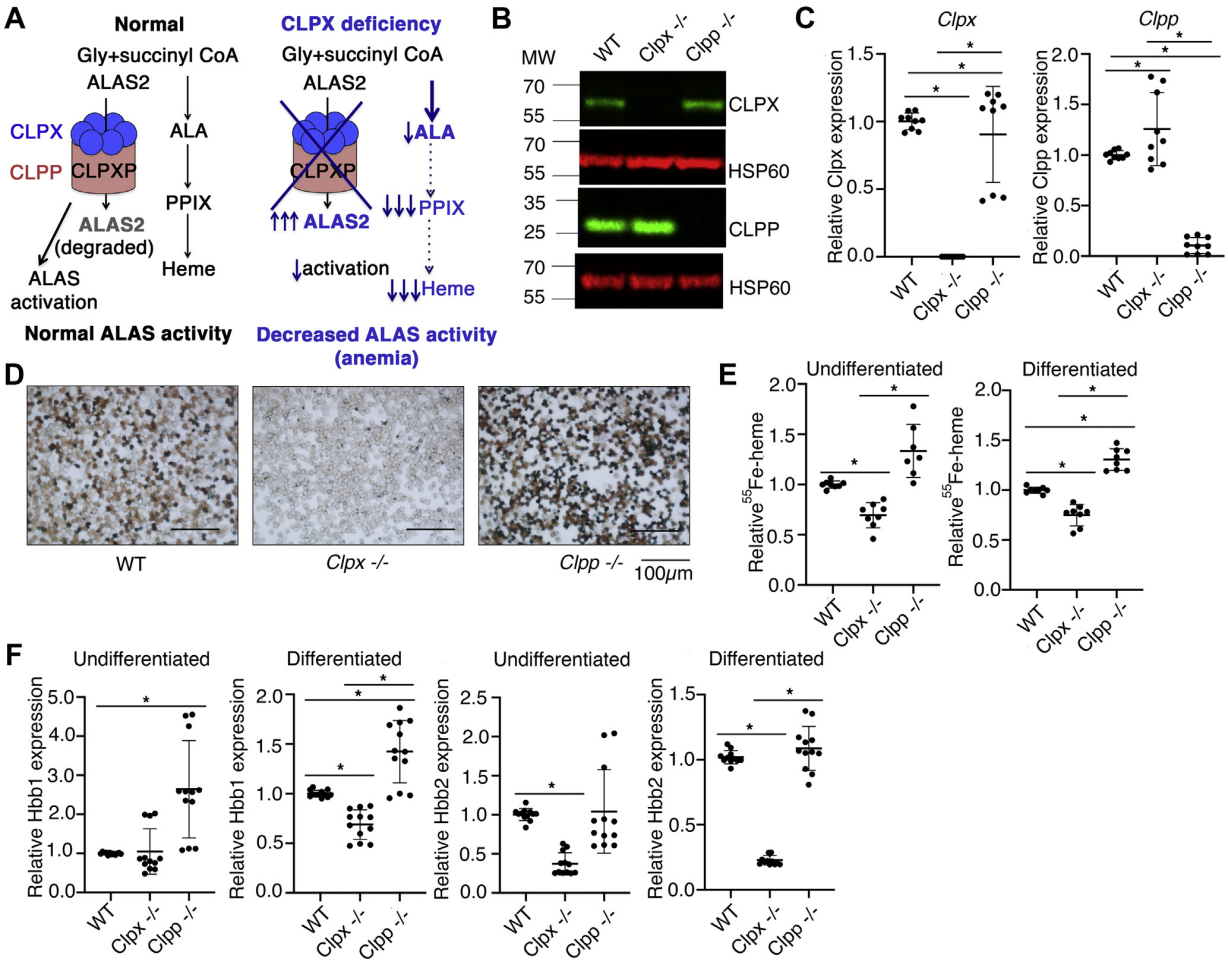
***Clpx***^***−/−***^**MEL cells have a heme synthetic defect distinct from the role of CLPX in the CLPXP protease.***A*, predicted model for the role of CLPXP in heme regulation. In yeast, CLPX activates the ALAS enzyme that catalyzes the committed step of heme synthesis. In mammalian cells, the CLPXP protease regulates ALAS stability and heme synthesis (16, 17). We hypothesized that CLPX-deficient erythroid cells will accumulate inactive ALAS protein due to defective activation by CLPX. Erythroid cells were predicted to be heme-deficient. *B*, Western blot analysis of CLPX and CLPP expression in WT, *Clpx*^*−/−*^, and *Clpp*^*−/−*^ MEL cell lysates (N = 4). HSP60 was used as a loading control. *C*, qPCR analysis of *Clpx* and *Clpp* mRNA expression in WT, *Clpx*^*−/−*^, and *Clpp*^*−/−*^ MEL cells. Gene expression was normalized to *β-actin* mRNA levels. *D*, benzidine staining of hemoglobin in *Clpx*^*−/−*^ and *Clpp*^*−/−*^ differentiated MEL cells. *Clpx*^*−/−*^ cells were hemoglobin-deficient, while *Clpp*^*−/−*^ cells produced more hemoglobin than WT cells. *E*, Quantitation of heme synthesis by ^55^Fe labeling. *Clpx*^*−/−*^ cells had a heme synthesis defect. In contrast, *Clpp*^*−/−*^ cells had increased heme synthesis. *F*, Differentiated *Clpx*^*−/−*^ cells had decreased *Hbb-b1* and *Hbb-b2* expression. Undifferentiated *Clpx*^*−/−*^ cells also had decreased *Hbb-b2* expression. ∗*p* < 0.05. Error bars indicate mean ± standard deviation (SD).

## Results

To determine the role of CLPX in erythroid heme synthesis, we conducted loss-of-function studies in murine erythroid cells. As CLPX-interacting regions of yeast ALAS (Hem1), required for CLPX to interact with and activate ALAS (18), had little similarity to vertebrate ALAS2 (Fig. S1), we wanted to determine if CLPX was required for ALAS2 activation in mammalian cells. We used CRISPR/Cas9 to knock out the *Clpx* and *Clpp* genes in mouse erythroleukemia (MEL) cells and verified the loss of CLPX and CLPP protein and mRNA expression by western blot (Fig. 1*B*) and qRT-PCR (Fig. 1*C*). Benzidine staining of differentiated *Clpx*^*−/−*^ and *Clpp*^*−*/*−*^ MEL cells indicated that *Clpx*^*−/−*^ cells were hemoglobin-deficient, consistent with published observations (Fig. 1*D*) (9). This was not caused by defects in mitochondrial activity (Fig. S2). *Clpp*^*−/−*^ cells, predicted to accumulate active ALAS2 due to defective CLPXP-catalyzed protein degradation, had increased hemoglobin (Fig. 1*D*). To quantify the role of *Clpx* and *Clpp* in heme synthesis, we labeled newly synthesized heme with ^55^Fe. *Clpx*^*−/−*^ MEL cells had decreased heme synthesis. In contrast, *Clpp*^*−/−*^ MEL cells synthesized increased quantities of heme (Fig. 1*E*). As heme is an important transcriptional regulator of globin (19, 20, 21, 22), we analyzed the effect of *Clpx* or *Clpp* deficiency on globin expression. Consistent with a heme defect in *Clpx*^*−/−*^ cells, differentiating *Clpx*^*−/−*^ cells experienced a significant decrease in *Hbb-b1* and *Hbb-b2* mRNA expression. Undifferentiated *Clpx*^*−/−*^ cells also had a significant decrease in *Hbb2* expression (Fig. 1*F*). These data confirm previous observations that CLPX is required for heme synthesis, while CLPP is not, and confirm that CLPX regulates heme independent of its role in the CLPXP proteolytic complex. *Clpx* deficiency also decreased globin mRNA expression, contributing to the hemoglobinization defect.

**Figure 2:**
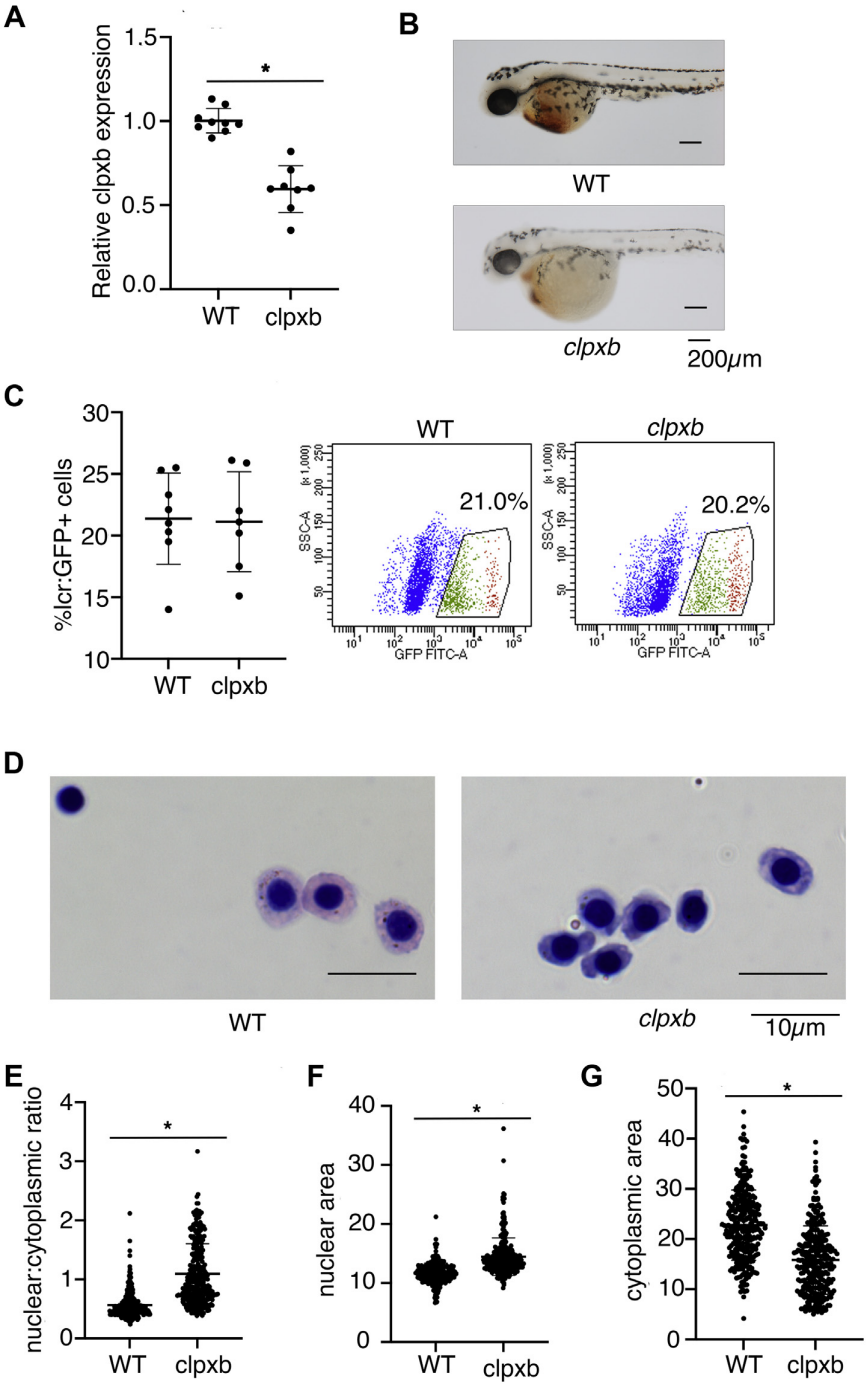
***clpxb* mutant zebrafish embryos develop normal numbers of red cells, but are heme-deficient and have dysregulated red cell morphology**. *A*, qPCR analysis of *clpxb* mRNA in WT and *clpxb* mutant embryos. *clpxb* mRNA is decreased by approximately 50% in mutant embryos (*p*-value < 0.0001). *B, clpxb* is required for erythroid hemoglobinization. *clpxb* mutant embryos are anemic at 48 hpf. *C, clpxb* is not required for erythroid specification. 72 hpf *clpxb/clpxb*; Tg(*lcr:GFP*) zebrafish embryos had similar percentages of GFP^+^ erythroid cells to control Tg(*lcr:GFP*) embryos as assessed by FACS. *D*, Compared with control erythroid cells, erythroid cells from *clpxb* mutant embryos were more variable in appearance, had smaller cytoplasmic areas and larger nuclei. *E, clpxb* erythroid cells had higher nuclear:cytoplasmic ratios (*p*-value < 0.0001). The nuclear:cytoplasmic ratios of *clpxb* erythroid cells exhibited higher variability (F test: *p* < 0.05). *F*, The increased nuclear:cytoplasmic ratio was caused by increased nuclear area in *clpxb* erythroid cells (*p*-value < 0.0001) and *G*, decreased cytoplasmic area (*p*-value<0.0001). ∗*p* < 0.05, Student's *t* test; error bars indicate SD.

To understand the role of *Clpx* in erythropoiesis *in vivo*, we characterized a zebrafish *clpxb* mutant (*clpxb*^*sa38141*^) obtained from the Zebrafish International Resource Center (ZIRC). Zebrafish encode two homologs of *Clpx*, *clpxa*, and *clpxb*. We previously investigated the role of the zebrafish *clpxa* gene in hematopoiesis, showing that *clpxa* deficiency caused a decrease in *gata1*-expressing erythroid progenitors and *globin-*expressing erythroid cells (9). As homologs in zebrafish arise by gene duplication, we wanted to determine if the *clpxb* homolog played redundant roles in erythropoiesis. The *clpxb*^*sa38141*^ mutant has an E129X nonsense mutation, which removes substantial portions of protein required for ATP binding. Homozygous mutant embryos express about half the *clpxb* mRNA as wild-type embryos due to nonsense mediated degradation (Fig. 2*A*). At 48 hpf, *clpxb* mutant embryos were anemic (Fig. 2*B*). To determine if the anemia resulted from erythroid differentiation defects, we quantitated erythroid cells in *clpxb* mutant *Tg*(*globin*-*lcr*:*GFP*) zebrafish (23). *clpxb* mutant embryos did not exhibit altered erythroid cell numbers, indicating that *clpxb* is not required for erythroid specification (Fig. 2*C*). Giemsa staining of sorted GFP^+^ cells revealed defects in *clpxb* erythroid cells, such as ruffled membranes and nonuniform morphology (Fig. 2*D*). In contrast to wild-type erythroid cells, *clpxb* mutant cells were larger and had more variable nuclear:cytoplasmic ratios (Fig. 2*E*) that were caused by increases in nuclear size (Fig. 2*F*) and decreases in cytoplasmic area (Fig. 2*G*). *clpxb* is hence required for erythroid heme synthesis and function but is not required for lineage determination or cell number.

**Figure 3:**
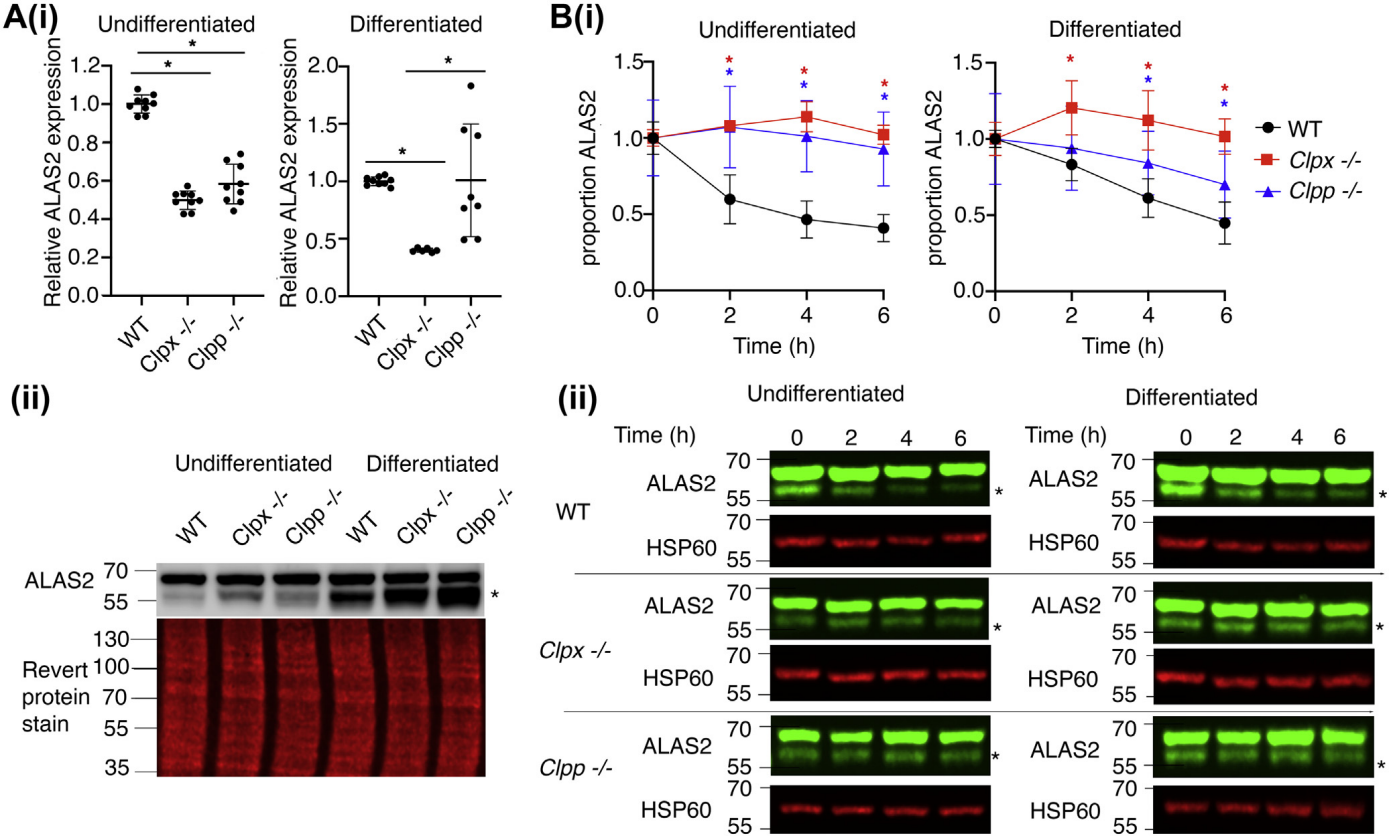
**CLPX regulates the stability of ALAS2 in MEL cells.***A*, *i*, *Alas2* mRNA expression was decreased or unaltered in the absence of CLPX and CLPP, (*ii*) but its protein levels are increased in *Clpx*^*−/−*^ and *Clpp*^*−/−*^ cells. The proteolytically cleaved, mitochondrial matrix localizing form of ALAS2 (38, 39, 40, 41, 42), which interacts with CLPX and catalyzes ALA formation (16), is marked with an asterisk (N = 3). *B*, ALAS2 stability is increased in both *Clpx*^*−/−*^ and *Clpp*^*−/−*^ cells. This is quantitated in (*i*), data shown relative to HSP60 expression. ∗*p* < 0.05. Error bars denote SEM (N = 4). Representative gels are shown in (*ii*).

To determine if CLPX regulates ALAS2, we analyzed the expression of its steady-state mRNA and protein. qPCR analysis revealed that *Alas2* mRNA decreased in undifferentiated *Clpx*^*−/−*^ and *Clpp*^*−/−*^ cells relative to wild-type cells. ALAS2 mRNA decreased in differentiated *Clpx*^*−/−*^ cells, relative to wild-type cells, but remained unchanged in *Clpp*^*−/−*^ cells (Fig. 3*A*, *i*). ALAS2 steady-state protein increased in both *Clpx*^*−/−*^ and *Clpp*^*−/−*^ cells (Fig. 3*A*, *ii*). These data suggested that CLPX and CLPP may regulate ALAS2 protein stability. In contrast to previous observations (24), we did not observe statistically significant decreases in SDHB protein levels or activity in *Clpx*^*−/−*^ and *Clpp*^*−/−*^ MEL cells (Fig. S3). To determine if CLPXP regulates ALAS2 stability, we monitored ALAS2 turnover in WT, *Clpx*^*−/−*^, and *Clpp*^*−/−*^ cells following inhibition of protein translation by cycloheximide (CHX) (17, 25, 26). ALAS2 was stabilized in *Clpx*^*−/−*^ and *Clpp*^*−/−*^ cells (Fig. 3*B*), echoing observations that CLPXP regulates ALAS1 turnover (16).

**Figure 4:**
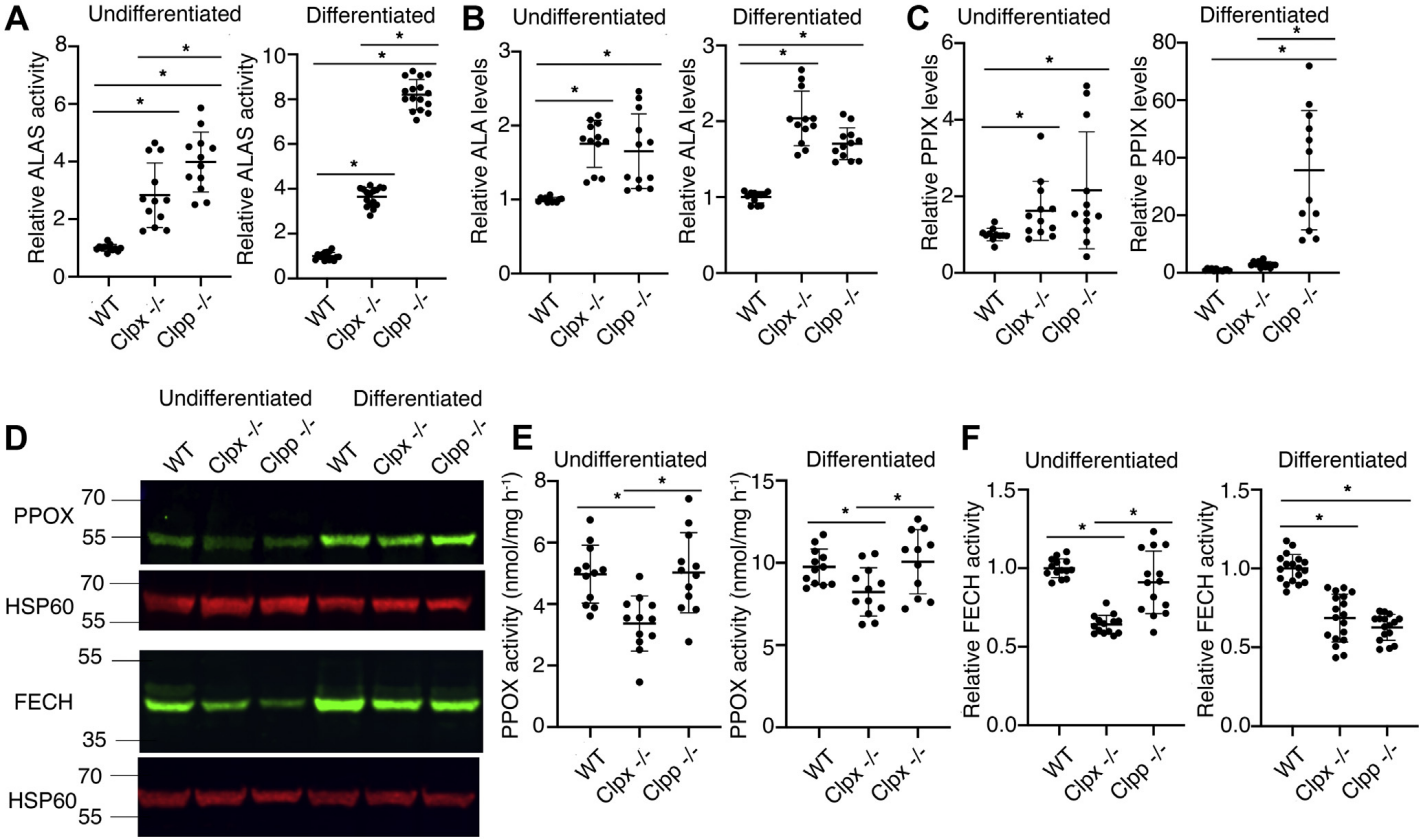
**CLPX regulates the terminal steps of heme synthesis.***A*, ALAS2 activity was significantly increased in *Clpx*^*−/−*^ and *Clpp*^*−/−*^ cells. *B, Clpx*^*−/−*^ and *Clpp*^*−/−*^ cells produced significantly more ALA than WT cells. *C, Clpx*^*−/−*^ and *Clpp*^*−/−*^ cells produced significantly more PPIX than WT cells. *D*, PPOX protein levels were decreased in undifferentiated *Clpx*^*−/−*^ and *Clpp*^*−/−*^ cells but not appreciably in differentiated cells. However, FECH protein levels were decreased, N = 3. *E*, PPOX activity was decreased in *Clpx*^*−/−*^ cells but was unaltered in *Clpp*^*−/−*^ cells. *F*, FECH activity was decreased in undifferentiated *Clpx*^*−/−*^ cells, relative to wild-type cells. The FECH activity of *Clpp*^*−/−*^ cells was unaltered. In differentiating MEL cells, FECH activity was decreased in both *Clpx*^*−/−*^ and *Clpp*^*−/−*^ cells. ∗*p* < 0.05. Error bars indicate SD.

ALAS activity assays showed that *Clpx*^*−/−*^ cells had significantly increased ALAS activity, indicating that CLPX is *not* required for ALAS activation (Fig. 4*A*). Strikingly, *Clpp*^*−/−*^ cells had even higher ALAS enzyme activity than *Clpx*^*−/−*^ cells. This dramatic increase in ALAS activity was most pronounced in differentiated *Clpp^−/−^* cells (Fig. 4*A*). The increase of ALAS activity in *Clpp*^*−/−*^ relative to *Clpx*^*−/−*^ cells cannot be attributed to differences in protein expression or stability (Fig. 3) suggesting that CLPP may play an inhibitory role in ALAS activation. An alternative explanation is that excess CLPX in *Clpp*^*−/−*^ cells (*i.e.*, CLPX that was not in a CLPXP complex) activated ALAS. The differential effect on ALAS activity did not translate to differences in ALA content as *Clpx*^*−/−*^ and *Clpp*^*−/−*^ cells had similar ALA levels (Fig. 4*B*). We quantitated PPIX, the terminal heme intermediate, to determine if porphyrin levels mirrored changes in ALA synthesis. Surprisingly, while *Clpx*^*−/−*^ and *Clpp*^*−/−*^ cells had similar increases in ALA levels, *Clpp*^*−/−*^ cells contained significantly more PPIX than *Clpx*^*−/−*^ cells. This was especially apparent in differentiated cells, suggesting that CLPXP may regulate heme synthesis downstream of ALA production (Fig. 4*C*). Hence, the heme defect in *Clpx*^*−/−*^ erythroid cells was not a result of a decrease in ALAS activation or porphyrin production.

Since CLPX is in the mitochondrial matrix (27), we hypothesized that CLPX may regulate the matrix localized terminal heme synthesis enzymes, PPOX and FECH (27) or mitochondrial iron metabolism (28). Western blot analysis showed that PPOX and FECH protein levels were slightly decreased in both knockout cell lines (Fig. 4*D*). However, only *Clpx*^*−/−*^ cells had a decrease in PPOX activity relative to WT or *Clpp*^*−/−*^ cells (Fig. 4*E*). FECH activity was decreased in *Clpx*^*−/−*^ cells in nondifferentiating cells. In *differentiating* cells, both *Clpx*^*−/−*^ and *Clpp*^*−/−*^ cells had decreased FECH activity (Fig. 4*F*). As *Clpp*^*−/−*^ cells did not exhibit a heme defect, it was unlikely that the FECH deficiency was the sole cause of the heme defect in *Clpx*^*−/−*^ cells.

**Figure 5:**
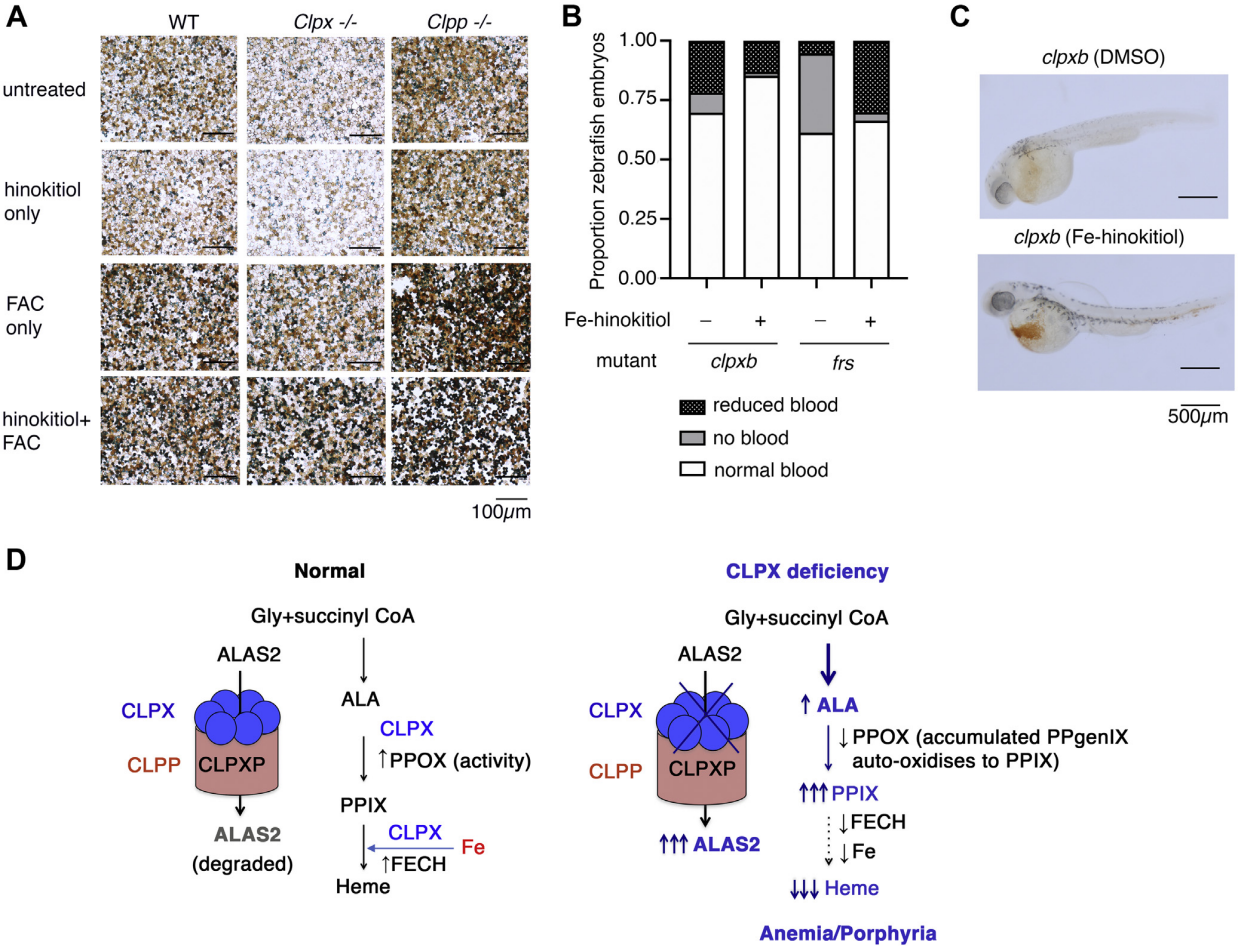
**CLPX regulates mitochondrial iron utilization in erythroid cells.***A*, iron supplementation restored hemoglobinization in *Clpx*^*−/−*^ cells. *B*, Fe-hinokitiol significantly reduced the numbers of anemic fish in *clpxb* incrosses. As a control, we treated *frs* incrosses with Fe-hinokitiol. Fe-hinokitiol significantly decreased the number of anemic fish and severity of anemia. *C*, representative images of benzidine stained *clpxb* zebrafish treated with Fe-hinokitiol. Erythroid hemoglobinization was significantly increased. *D*, revised model of how CLPX regulates erythroid heme synthesis. CLPX regulates ALAS2 turnover. In its absence, ALAS2 protein levels and activity are increased, increasing ALA levels. Concomitantly, PPOX and FECH activities, and iron incorporation into heme, are decreased, resulting in anemia and porphyria during CLPX deficiency.

We also considered the possibility that CLPX regulated mitochondrial iron utilization. We chemically complemented the heme defect in *Clpx*^*−/−*^ cells with ferric ammonium citrate complexed with a lipophilic iron chelator, hinokitiol (29). Treatment with Fe-hinokitiol increased heme content in *Clpx*^*−/−*^ cells to levels close to wild-type. These results suggested that a mitochondrial iron defect contributed to the heme defect in *Clpx*^*−/−*^ cells (Fig. 5*A*). We also treated zebrafish embryos spawned from *frs* or *clpxb* heterozygous incrosses with either vehicle (DMSO) or Fe-hinokitiol. *frs* mutant zebrafish lack expression of *mfrn1*, the mitochondrial iron transporter (28, 30) and served as a positive control for this experiment as their anemia was ameliorated by Fe-hinokitiol (29). As expected, the numbers of severely anemic zebrafish embryos in *frs* incrosses were sharply reduced when treated with Fe-hinokitiol. Fe-hinokitiol significantly decreased numbers of embryos from *clpxb* heterozygote incrosses in both “severely anemic” and “anemic” categories, but the results were not as clear-cut as the *frs* mutant zebrafish (Fig. 5, *B* and *C*). These intermediate results indicate that iron deficiency plays a role in anemia in the *clpxb* mutant embryos (Fig. 5, *B* and *C*), but is not the sole cause of the anemia. Neither *Clpx*^*−/−*^ nor *Clpp*^*−/−*^ cells had a defect in MFRN1 expression (Fig. S4*A*) or mitochondrial iron levels (Fig. S4*B*), suggesting the defect is not in mitochondrial iron transport.

Our results indicate that the primary mechanism by which CLPX regulates ALAS in vertebrate cells is by control of its turnover, rather than its activity. We have also shown that CLPX is required for maximal PPOX and FECH activity and regulates mitochondrial iron metabolism in differentiating erythroid cells. The porphyrin accumulation observed in these cells is augmented by increased ALAS2 activity in these cells (Fig. 5*D*).

## Discussion

Although the requirement for *Clpx* in heme synthesis is conserved from yeast to vertebrates, several differences in CLPX function exist. The role of CLPX in heme regulation was originally discovered in *S. cerevisiae* yeast, which do not possess a CLPP ortholog. In yeast, CLPX functions exclusively as a mitochondrial protein unfoldase that unfolds Hem1/ALAS, facilitating the incorporation of its PLP cofactor, leading to ALAS/Hem1 activation (9). Vertebrates, however, possess a CLPP ortholog, which forms the CLPXP protease with CLPX. In vertebrate cells, CLPXP regulates ALAS turnover (16). Previous studies on vertebrate CLPX did not examine the requirement of CLPX for ALAS activity or heme synthesis. Our current studies bridged this gap by assaying the effects of *Clpx* and *Clpp* loss of function on the activities of heme synthetic enzymes and ALAS2 stability, as CLPX was previously shown to be required for erythroid hemoglobinization in zebrafish (9). *Clpx*^*−/−*^ erythroid cells have elevated ALA content and ALAS activity, unlike in yeast. In contrast to previous predictions in yeast, the heme defect in *Clpx*^*−/−*^ erythroid cells was not due to defects in ALAS activity (Fig. 4, *A* and *B*). However, ALAS activity in *Clpp*^*−/−*^ cells is higher than that of *Clpx*^*−/−*^ cells even though its steady-state protein levels in *Clpx*^*−/−*^ and *Clpp*^*−/−*^ cells are similar (Fig. 3*A*). This suggests that CLPX may play a nonessential role in ALAS activation, leading to *Clpp*^*−/−*^ cells exhibiting a “superactivated” ALAS phenotype as CLPX activates accumulated ALAS2.

PPIX content was elevated in *Clpx*^*−/−*^ and *Clpp*^*−/−*^ cells, caused by a combination of elevated ALA production with downstream heme synthesis and iron metabolism defects (Fig. 4*C*). *Clpx*^*−/−*^ cells had decreased PPOX and FECH activity, partially accounting for the heme defect (Fig. 4, *E* and *F*). The increase in PPIX levels was due to increased protoporphyrinogen IX (PPgenIX) caused by ALA overproduction and defects in PPOX and FECH activity. PPgenIX auto-oxidises to PPIX (31) and leads to increased measurable PPIX even with decreased PPOX activity in *Clpx*^*−/−*^ cells. In differentiating MEL cells, FECH activity was decreased in *Clpp*^*−/−*^ cells, which exacerbated PPIX accumulation, although this did not cause heme deficiency. The heme defect in *Clpx*^*−/−*^ cells was exacerbated by an iron defect, which was ameliorated by exogenous iron (Fig. 5, *A* and *B*).

While CLPX is best known for its proteolytic function (11), it has roles that are distinct from CLPXP-mediated proteolysis. Our data support the premise that CLPX has CLPXP-independent functions in heme synthesis. While PPOX protein levels are slightly decreased in *Clpx*^*−/−*^ and *Clpp*^*−/−*^ MEL cells, PPOX activity was decreased in only *Clpx*^*−/−*^ cells, suggesting that CLPX plays a role in PPOX activation. Further, iron complementation studies suggest that *Clpx*^*−/−*^, but not *Clpp*^*−/−*^, erythroid cells have an iron metabolism defect. Our studies suggest that CLPX regulates the function of mitochondrial matrix proteins that regulate heme synthesis in erythroid cells.

The interactions between CLPX, CLPP, and erythroid heme synthesis are complex and are likely to be tissue and developmental stage specific. Several CLPXP-binding proteins are iron–sulfur (Fe-S) cluster proteins that are involved in oxidative phosphorylation or Fe-S cluster assembly (32). A mouse model of Friedreich ataxia with a striated-muscle specific frataxin knockout showed an increase in expression of the CLPP and LON proteases associated with a decrease in mitochondrial Fe-S proteins. These included FECH, NDUFS3, and SDHB, which are involved in heme synthesis and oxidative phosphorylation (33). These data contrast with the decrease in FECH activity that we observed in our *Clpx*^*−/−*^ and *Clpp*^*−/−*^ erythroid cell lines, although the decreased FECH activity may be caused by defects in iron metabolism in *Clpx*^*−/−*^ cells (Fig. 4, *A* and *C*). Unlike previous studies on prostate adenocarcinoma cells (24), SDHB protein levels and activity were relatively unchanged in *Clpx*^*−/−*^ and *Clpp*^*−/−*^ MEL cells (Fig. S3). Lastly, CLPXP interacts with and degrades ALAS1 in the presence of heme, but dissociates from ALAS1 when heme synthesis is suppressed; in contrast, ALAS2 turnover is not increased during erythroid differentiation, when there are increased levels of heme (Fig. 3*B*). This suggests that unlike ALAS1, CLPXP does not increase the degradation of ALAS2 under high heme conditions. It is likely that the specific roles of CLPXP are regulated by cell-specific proteins. During terminal differentiation, the function of erythroid mitochondria shifts toward heme production for hemoglobin synthesis (8, 34, 35, 36, 37). It is therefore revealing that CLPXP regulates heme synthesis at multiple points in the pathway, but is not essential for regulating Fe-S proteins in erythroid cells.

Our data shed light on how a dominant *CLPX*^*G298D*^ heterozygous mutation caused erythroid protoporphyria (17). CLPX^G298D^ lacked ATPase activity and hetero-oligomerized with wild-type CLPX, decreasing the ATPase activity of the CLPX hexamer. We previously proposed that the WT/G298D hetero-hexamer could activate, but not degrade ALAS, causing accumulation of active ALAS and overproduction of PPIX, leading to EPP. Our new data indicate that CLPX is not required for activation of ALAS, suggesting that CLPX-EPP can be caused by stabilization of ALAS alone. We also show that CLPX and CLPP are required for optimal FECH activity. The decrease in FECH activity can exacerbate PPIX accumulation caused by ALA overproduction and may be compounded by an iron defect.

CLPX mutations contribute to metabolic disorders in human patients and animal models (12, 13, 17, 33), and CLPX regulates heme synthesis in erythroid cells by control of mitochondrial heme synthesis and iron utilization. More broadly, heme regulation is not limited to control of ALAS activity, long regarded as the rate-limiting step. Rather, it can also be controlled at the terminal steps, *i.e.*, at the level of PPOX and FECH activity, and their integration with iron utilization. The heterogeneity of CLPX-deficient phenotypes in different cell types suggests that CLPX regulates mitochondrial metabolism in a cell-specific manner (9, 15, 24, 32). We propose that CLPX interacts with cell-specific factors to couple mitochondrial metabolism with cellular requirements. Unraveling the complexities of CLPX function will be key for designing therapies for metabolic diseases and mitochondriopathies.

## Experimental procedures

Methods are provided in the supporting information.

### Vertebrate animal study approval

Vertebrate animal studies were performed in compliance with Institutional Animal Care and Use Committee protocols at the University of Delaware.

## Data availability

All data are contained in the manuscript.

## Supporting information

This article contains supporting information (8, 17, 29, 36).

## Conflict of interest

The authors declare that they have no conflicts of interest with the contents of this article.
