# Supplementary material for: The ubiquitous mitochondrial protein unfoldase CLPX regulates erythroid heme synthesis by control of iron utilization and heme synthesis enzyme activation and turnover
Source: J Biol Chem. 2021 Jul 16;297(2):100972. doi: 10.1016/j.jbc.2021.100972 (PMC8361296; doi:10.1016/j.jbc.2021.100972)
Supplement: Supporting information [file mmc1.pdf]

Supporting Information

The ubiquitous mitochondrial protein unfoldase CLPX regulates erythroid heme synthesis by control of iron utilization and heme synthesis enzyme activation and turnover

Catherine M. Rondelli[1*], Mark Perfetto[1,2*], Aidan Danoff[1*], Hector Bergonia[3], Samantha Gillis[1], Leah O'Neill[1], Laurie Jackson[3], Gael Nicolas[4], Herve Puy[4,5], Richard West[6], John D. Phillips[3], Yvette Y. Yien[1,2]

[1] Department of Biological Sciences, University of Delaware, Newark, Delaware 19716.
[2] Pittsburgh Heart, Lung and Blood Vascular Medicine Institute and Department of Medicine, University of Pittsburgh, Pittsburgh, PA 15261, USA
[3] Division of Hematology, University of Utah School of Medicine, Salt Lake City, Utah 84112
[4] Centre de Recherche sur l'inflammation, Université Paris Diderot, Site Bichat, Sorbonne Paris Cité, 75018 Paris, France.
[5] Centre Français des Porphyries, Hôpital Louis Mourier, APHP, 92701 Colombes, France.
[6] Delaware Biotechnology Institute, University of Delaware, Newark, Delaware 19713.

Address correspondence to: Yvette Y. Yien, Department of Biological Sciences, University of Delaware. 105 The Green, Newark, DE 19716, USA; Pittsburgh Heart, Lung and Blood Vascular Medicine Institute and Department of Medicine, University of Pittsburgh.
1200 Biomedical Science Tower, 200 Lothrop Street, Pittsburgh, PA 15261, USA
Email: yyien@udel.edu; yieny@pitt.edu.

*Authorship note: Catherine Rondelli, Mark Perfetto and Aidan Danoff contributed equally to this work.

Supplemental Methods

Vertebrate animal study approval

Vertebrate animal studies were performed in compliance with Institutional Animal Care and Use Committee protocols at the University of Delaware.

Zebrafish lines

The *clpxb*^{sa38141} zebrafish mutant line was obtained from ZIRC. Zebrafish were genotyped by PCR amplifying the region around the *clpxb* mutation with these primers: Fwd 5'- ACTGGAGAAGAGCAACATTGTGTTG -3' and Rev 5'- CGATGTCTTCGCCCACATATCC - 3'. The mutation was confirmed by direct Sanger sequencing of the PCR product.

Functional complementation with iron

MEL cells were differentiated with 2% DMSO for 72 h. For functional complementation assays with hinokitiol, cells were concurrently treated with 10 μM ferric ammonium citrate and 1 μM hinokitiol. Zebrafish embryos were treated with 1μM hinokitiol and 10 μM ferric citrate (1). In total, 935 embryos in 4 clutches were scored.

Iron radiolabeling and radio iron-heme measurements

$^{55}FeCl_3$ (Perkin Elmer) was loaded onto transferrin as previously described (2). Metabolic labeling with ^{55}Fe-transferrin and quantitation of ^{55}Fe-heme were carried out as previously described (3).

Western analysis and validation of antibodies

The following primary antibodies and dilutions were used in this study: ALAS2 1:1000 (Abcam, ab184964) (4,5) which detected a doublet, between 55-70 kDa, which increased in intensity in differentiated MEL cells and in *Clpx -/-* and *Clpp -/-* cells. The increase in ALAS2 protein levels during differentiation correlated with the increase in ALAS2 mRNA during differentiation (Supplemental Figure 5). FECH 1:500 (EMD Millipore Corp, ABS2124) which detected a single band between 33-55 kDa that increased in intensity in differentiated MEL cells was detected (Figure 4). PPOX 1:500 (Abnova, H00005498-M01) (6) which detected a single band at about 55 kDa that increased in intensity in differentiated MEL cells was detected (Figure 4). CLPX 1:1000 (Abcam, ab168338), CLPP (Abcam, ab12482) 1:1000 were validated in Figure 1. MFRN1 (Proteintech, 26469-1-AP), 1:500 was previously published and detected a band that increased in intensity during G1ER erythroid cell differentiation (7). SDHB (Abcam ab14714), 1:1000 was used as previously published (8) Other antibodies used to blot for OXPHOS proteins were: ATP5A 1:1,000 (Abcam ab14748), SDHB 1:1,000 (Abcam ab14714), UQRC2 1:1,000 (Abcam ab14745), COX IV 1:1,000 (Abcam ab202554), NDUFB8 1:1,000 (Abcam ab110242). HSP60 1:750 (Abcam ab59457), HSP60 1:750 (Sigma, A5316) and β-actin 1:1000 (Santa Cruz, sc47778) were used as loading controls. Fluorescent secondary antibodies (LI-COR) were used at a dilution of 1:10,000. Secondary antibodies for chemiluminescence (Sigma) were used at a dilution of 1:10,000.

qRT-PCR

Taqman probes were obtained from Invitrogen. To quantitate zebrafish *clpxb* mRNA, we designed a probe (5' CTGGGACCCACTGGATCAGGTAAAACTCTCCTGGCCCAAACA 3')

which bound between exons 8-9. Actin was used as a normalization control (Dr03432610_m1). For mouse cell lines, the following probes were used: *Alas2* Mm00802083_m1; *Clpx* Mm00488586_m1; *Clpp* Mm00489940_m1; *β-actin* Mm02619580_g1; *Hbb-b1* Mm01611268_g1; *Hbb-b2* Mm00731743_mH.

A TaqMan custom FAM assay was used for *clpxb* expression analysis. The clpxb primers were generated spanning exon 8 and exon 9: exon 8 5' ACTGGAGAAGAGCAACATTGTGTTG 3' and exon 9 5' CGATGTCTTCGCCCACATATCC 3'. The probe sequence was 5' CTGGGACCCACTGGATCAGGTAAAACTCTCCTGGCCCAAACA 3'.

Cycloheximide block

Inhibition of translation was carried out with 100 μg/mL cycloheximide (Sigma-Aldrich) as previously described (5,9). Cells were harvested at indicated times after CHX treatment. Protein from whole cell lysates were resolved by SDS/PAGE, detected by Western blot analysis, and quantified using a LI-COR imager.

Single embryo cell extraction and FACS sorting of GFP$^+$ erythroid cells

Embryos were anesthetized tricaine and individual embryos were placed on ice prior to processing and materials and solutions were chilled to maintain integrity. Embryos were rinsed with PBS, crushed thoroughly with a tube pestle, and remaining tissue mechanically dissociated. All remaining steps were done on ice. The cell solution was filtered, rinsed with PBS. Cells were collected by centrifugation and resuspended in 0.05% gluteraldehyde fixative. Fixed cells were collected by centrifugation and rinsed with PBS and PBS + 0.1%BSA. Cells were sorted using a FACSaria Fusion at 600 cells per slide. Unsorted cells were genotyped.

Generation of CRISPR cell lines

CRISPR guide sequences were designed to direct two cleavages at the target gene loci to generate a chromosomal deletion (10,11). CRISPR guide sequences were designed to have a unique 12 bp seed sequence 5'-NNNNNNNNNNNN-NGG-3' in the mouse genome (http://www.genome-engineering.org) to minimize off-target cleavages. We targeted exons 3 and 11 of the *Clpx* gene with the following sequences: 5'CACCGAAGGAAGCAGTAAGAAATCG 3' (exon 3) and 5' CACCGGATCTTGCTAACCGAAGTGG 3' (exon 11). We targeted exons 1 and 4 of the *Clpp* gene with the following sequences: 5' CACCGAGGCCCGGGTGGCTGTGGAG 3' (exon 1) and 5' CACCGTTGGACAGGCTGCCAGCATG 3' (exon 4). CRISPR guides were cloned into pX330 plasmid (Addgene) with BbsI ligation as previously described (12).

CRISPR/Cas9 constructs were delivered to mouse erythroleukemia (MEL) cells by electroporation using a Biorad electroporator. CRISPR/Cas9 constructs were co-electroporated

with pEF1α at a 9:1 ratio. Cell selection was carried out as previously described (13). Clones were screened for loss of CLPX or CLPP protein by western analysis and qRT PCR.

Biochemical analyses
Cells were spun down, washed with PBS at 4°C, and flash frozen for shipping to the University of Utah on dry ice. Activities of the ALAS, FECH and PPOX enzymes, and sample ALA and PPIX levels were measured at the Iron and Heme Core at the University of Utah. ALAS, FECH and PPOX enzyme activity assays, and ALA and PPIX levels are quantified as described:

PPOX enzyme activity assay (14)
PPgenIX substrate was prepared by reduction of PPIX. 1mM PPIX was prepared by dissolving in an aqueous solution of 10mM KOH and 20% v/v ethanol. 150 mg of 20% sodium in mercury amalgam was introduced into the PPIX solution and stirred under blowing argon gas until the fluorescence was gone (as monitored by brief exposure to a UV lamp). After the reaction was complete, the solids were removed with a syringe filter. 9 vol of incubation buffer (120mM Tris base, 2.5mM EDTA free acid, 100mM ascorbic acid and 0.12% v/v Triton X-100. pH of PPgenIX solution was adjusted to 8.5 with 3M HCl before use.

Cell pellets were resuspended in about 3 volumes of 50 mM potassium phosphate, pH 7.4 and sonicated for 3 cycles x 5s at 50% duty (5s on and 5s off). Protein concentration was adjusted to 4 mg/ml with 50mM potassium phosphate.

For the PPOX enzyme activity assay, 90μl of PPgenIX substrate was added to 10μl of sample containing 4 mg/ml protein and incubated at 37°C for 10 min. The reaction was stopped by adding 400μl of 30% v/v DMSO in ethanol. The reaction was centrifuged at 16,000g for 10 min and the supernatant was injected into a Waters Acquity ultra performace liquid chromatography system (UPLC). The PPIX product was quantified with a fluorescence detector set at 404 nm excitation and 630 emission to measure fluorescence of PPIX. Known concentrations of PPIX dissolved in 30% v/v DMSO in ethanol were used as quantification standards.

ALAS activity assay and ALA quantification (15)

Washed and packed cell pellets were resuspended about three volumes of 50 mM potassium phosphate (KPi) pH 7.4. The suspension was homogenized by a brief sonication using a Sonicare W-380 Ultrasonic Processor (Heat Systems - Ultrasonics, Inc., Farmingdale, NY), while in an ice bath. The protein content was measured by the Pierce BCA Protein Assay Method (Thermo Scientific, Rockford, IL) and using a SpectraMax 190 microplate reader paired with SoftMax Pro v5.0b7 software, both from Molecular Devices Corp (Sunnyvale, CA). About 25 μL of homogenate containing 10 mg/mL protein was mixed with 25 μL of assay buffer that contained 0.1 M glycine pH 7, 1 mM succinylCoA, 160 μM pyridoxal 5'-phosphate and 0.1 mM succinylacetone. The resulting mixture was incubated at 37 °C for 30 min, and then 450 μL of ice-cold water was added to stop the reaction. Fifty μL of the stopped reaction was mixed with 150 μL derivatization agent that consisted of water, 37% formaldehyde, ethanol and acetylacetone in a ratio of 107:5:15:23 by volume, respectively. The resulting solution was incubated at about 100–103 °C for 5.0 min, cooled immediately in an ice bath for at least 1 h in the dark, and then centrifuged for 10 min at 16000xg in a microfuge at 4 °C.

The supernatant was analyzed by ultra-performance liquid chromatography (UPLC). Ten μL of supernatant containing the derivatized ALA was injected into a Waters Acquity UPLC system which included a binary solvent manager, sample manager, fluorescence detector, column heater and an Acquity UPLC BEH C18, 1.7 μM, 2.1 Å 100 mm column. The fluorescence detector was set at 370 nm excitation and 460 nm emission, while the sample chamber was kept dark and at 5 °C. Solvent A was 0.2 % aqueous formic acid while Solvent B was 100 % methanol. The flow rate was constant at 0.3 mL/min and the column at 50°C for the total run time of 12 min. The following gradient schedule with the percent Solvent A at each step was as follows: 0 min, 80%; 6 min, 60%; 7 min, 1%; 9 min, 1%; and 9.5 min, 80%. The gradient for solvent composition from 0 to 6 min was set at Waters Gradient 5 (convex, with a higher slope at 0 min compared to that at 6 min), and that from 6 to 7 min at Waters Gradient 7, concave. All other gradients in the method were linear.

Blanks were samples heated in boiling water a bath for 10 min. Standard curves were constructed using samples spiked with known amounts of ALA.

Endogenous ALA in the samples was quantified by mixing 50μL of homogenate with 150μL of derivatization agent as described above, and analyzed similarly.

PPIX quantification (16)
Sample homogenates were prepared as described for the ALAS assay and diluted with KPi to contain 10 mg protein/mL. The extraction procedure was adapted from Peter et al. (1978)(54). Fifty μL of the adjusted sample was slowly mixed with 200 μL extracting solvent (EA) while being vortexed vigorously, and then agitated further for another 60 sec. EA contained four volumes of ethyl acetate and one volume of glacial acetic acid. The extraction mixture was centrifuged at 16000xg for 0.5 min and the supernatant was collected and analyzed by UPLC.

About 10μL of the supernatant solution above was injected into a Waters Acquity UPLC system which included a binary solvent manager, sample manager, photodiode array detector (PDA), fluorescence detector (FLR), column heater and an Acquity UPLC BEH C18, 1.7 μM, 2.1 x 100 mm column. The FLR was set at 404 nm excitation and 630 nm emission to measure fluorescence of protoporphyrin IX (PPIX). The PDA was used to verify the absorption spectrum of PPIX. The sample chamber was kept dark and at ambient temperature. Solvent A was 0.2% aqueous formic acid while Solvent B was 0.2% formic acid in methanol. The flow rate was kept at 0.40 mL per minute and the column maintained at 60˚C for the total run time of 7 min. The following successive linear gradient settings for run time in minutes versus Solvent A were as follows: 0.0, 80%; 2.5, 1%; 4.5, 1%; 5.0, 80%. For standards, solutions of known concentrations of PPIX dissolved in 1% aqueous trimethylamine were extracted similarly.

Ferrochelatase activity assay (17)
Ferrochelatase activity was measured as described (55)) with some modifications. Washed and packed cell pellets were mixed with four volumes of tris buffered glycerol with DTT (TGD, made by dissolving 2 mL and 1.5 mg DTT in 8.0 mL 20 mM Tris pH 8.0). The suspension was sonicated using a Sonicare W-380 Ultrasonic Processor (Heat Systems - Ultrasonics, Inc., Farmingdale, NY). The resulting homogenate was assayed for protein using a Pierce BCA

protein assay kit (Thermo Sicentific, Rockford, IL) and a SpectraMax 190 microplate reader paired with SoftMax Pro v5.0b7 software, both from Molecular Devices Corp (Sunnyvale, CA). The samples were diluted to 1 µg protein/µL with more TGD before ferrochelatase measurement.

A 50-µL sample aliquot was mixed with 150µL incubation buffer (160 mM Tris pH 8.0, 40 mM Bicine pH 8.0, 10 mg/ml Tween 20 and 0.38 mg/mL palmitic acid), plus 25 µL zinc substrate (1mM aqueous Zn acetate) and pre-incubated for 5 minutes at 37˚C. Then 25 µL of mesoporphyrin IX substrate (250 µM in 160 mM Tris pH 8.0, 40 mM Bicine pH 8.0, 2 mg/ml Tween 20) was added and the mixture was incubated for 30 min at 37˚C. The reaction was stopped with 750 µL of DMSO-methanol-EDTA mixture (270 µM EDTA in DMSO-methanol, 30/70 by volume), cooled on ice for 15-20 min, and then centrifuged at 1500xg for 10 min at room temperature.

Ten microliters of supernatant was injected into a Waters (Millford, MA) Acquity ultra-performance liquid chromatography (UPLC) system that consisted of a binary solvent manager, sample manager, column heater, a photodiode array (PDA) detector and an Acquity UPLC BEH C18, 1.7 µM, 2.1 x 100 mm column. The fluorescence detector was optimized for zinc mesoporphyrin IX at 406 nm excitation and 578 nm emission, and the ferrochelatase product was quantified relative to a standard solution, also in the DMSO-methanol-EDTA mixture. Blanks were samples heated in boiling water a bath for 10 min. The product Zn mesoporphyrin IX was resolved using a solvent gradient that consisted of A, 0.2% v/v formic acid in water, and B, 0.2% formic acid in methanol. Flow rate was 0.4 mL/min and the column was set at 60˚C, for the total run time of 7 min. The following successive linear gradient settings were used for run time in minutes versus A: 0.0, 80%; 2.5, 1%; 4.5, 1%; 5.0, 80%.

ICP-MS (inductively coupled plasma mass spectroscopy) metal analysis

Mitochondria were isolated with a kit from Thermo (Cat#: 89874). Mitochondrial pellets were frozen on dry ice and shipped to the Center of Iron and Heme Disorders at the University of Utah.

Sample pellets were resuspended in distilled water and sonicated. Protein concentration was determined using BCA Protein Assay (Thermo Scientific). Lysed sample aliquots were digested using a 5:1 mixture of OPTIMA nitric acid and ultrapure hydrogen peroxide (Fisher Scientific). This mixture is incubated at room temperature overnight, heated at 95°C until dry, and resuspended overnight in 2% nitric acid prior to analysis. An Agilent 7900 ICP-MS was operated in helium (He) collision cell gas mode for all measurements. Elements are measured at the following isotopes: ^{56}Fe, ^{55}Mn, ^{63}Cu, ^{66}Zn, ^{72}Ge, ^{45}Sc. Calibration standards and samples are prepared in an acid matrix of 2% OPTIMA Grade Nitric Acid. Solutions of Agilent Multi-element Calibration Standard 2A are prepared to obtain an eight-point calibration curve. Agilent Germanium and Scandium Standards are added to calibration standards, blanks and samples, and are used to correct for potential sample matrix and/or nebulization effects.

Supplemental figure legends

Figure 1

B, Change from undifferentiated WT (±SEM): CLPX: *Clpx*$^{-/-}$ =ND (because of undetectable CLPX signal) and *Clpp*$^{-/-}$ =1.714±0.455; CLPP: *Clpx*$^{-/-}$ =1.768±0.132 and *Clpp*$^{-/-}$ = ND (not detectable)
C, P-values: CLPX: WT vs *Clpx*$^{-/-}$ and *ClpX*$^{-/-}$ vs *Clpp*$^{-/-}$ <0.001; CLPP: WT vs *Clpx*$^{-/-}$ =0.0384, WT vs *Clpp*$^{-/-}$ and *Clpx*$^{-/-}$ vs *Clpp*$^{-/-}$<0.0001.
E, P-values: Undifferentiated: WT vs *Clpx*$^{-/-}$ =0.0058, WT vs *Clpp*$^{-/-}$ = 0.0038, and *Clpx*$^{-/-}$ vs *Clpp*$^{-/-}$ <0.0001; Differentiated: WT vs *Clpx*$^{-/-}$ =0.0003, WT vs *Clpp*$^{-/-}$ and *Clpx*$^{-/-}$ vs *Clpp*$^{-/-}$ <0.0001.
F, P-values: *Hbb-b1*: Undifferentiated: WT vs *Clpx*$^{-/-}$ =0.0006 and *Clpx*$^{-/-}$ vs *Clpp*$^{-/-}$ =0.0009; Differentiated: WT vs *Clpx*$^{-/-}$ =0.0008, WT vs *Clpp*$^{-/-}$ =0.0042, and *Clpx*$^{-/-}$ vs *Clpp*$^{-/-}$ <0.0001. *Hbb-b2*: Undifferentiated: WT vs *Clpx*$^{-/-}$ =0.0004, *Clpx*$^{-/-}$ vs *Clpp*$^{-/-}$ =0.0002; Differentiated: WT vs *Clpx*$^{-/-}$ and *Clpx*$^{-/-}$ vs *Clpp*$^{-/-}$ <0.0001.

Figure 3

A, P-values: Undifferentiated: WT vs *Clpx*$^{-/-}$ and WT vs *Clpp*$^{-/-}$<0.0001, *Clpx*$^{-/-}$ vs *Clpp*$^{-/-}$ =0.0171; Differentiated: WT vs *Clpx*$^{-/-}$ = 0.0077 and *Clpx*$^{-/-}$ vs *Clpp*$^{-/-}$ = 0.0072

Figure 4

A, P values: Undifferentiated: WT vs *Clpx*$^{-/-}$ and WT vs *Clpp*$^{-/-}$ <0.0001, *Clpx*$^{-/-}$ vs *Clpp*$^{-/-}$ = 0.0001; Differentiated: WT vs *Clpx*$^{-/-}$, WT vs *Clpp*$^{-/-}$, and *Clpx*$^{-/-}$ vs *Clpp*$^{-/-}$ <0.0001.
B, P values: Undifferentiated: WT vs *Clpx*$^{-/-}$ and WT vs *Clpp*$^{-/-}$ <0.0001; Differentiated: WT vs *Clpx*$^{-/-}$ and WT vs *Clpp*$^{-/-}$ <0.0001, and *Clpx*$^{-/-}$ vs *Clpp*$^{-/-}$ =0.0091.
C, P values: Undifferentiated: WT vs *Clpp*$^{-/-}$ = 0.0172; Differentiated: WT vs *Clpp*$^{-/-}$ and *Clpx*$^{-/-}$ vs *Clpp*$^{-/-}$ <0.0001.
D, PPOX protein levels were decreased in undifferentiated *Clpx*$^{-/-}$ and *Clpp*$^{-/-}$ cells (Fold change from Undifferentiated WT (±SEM): *Clpx*$^{-/-}$=0.583±0.096 and *Clpp*$^{-/-}$ =0.632±0.048) but not appreciably in differentiated cells (Differentiated WT: *Clpx*$^{-/-}$ =0.850±0.056 and *Clpp*$^{-/-}$ =1.307±0.145). However, FECH protein levels were decreased. (Fold change from Undifferentiated WT (±SEM): *Clpx*$^{-/-}$ =0.855±0.081 and *Clpp*$^{-/-}$ = 0.587±0.074; from Differentiated WT: *Clpx*$^{-/-}$ = 0.575±0.039 and *Clpp*$^{-/-}$ = 0.595±0.094), N=3.

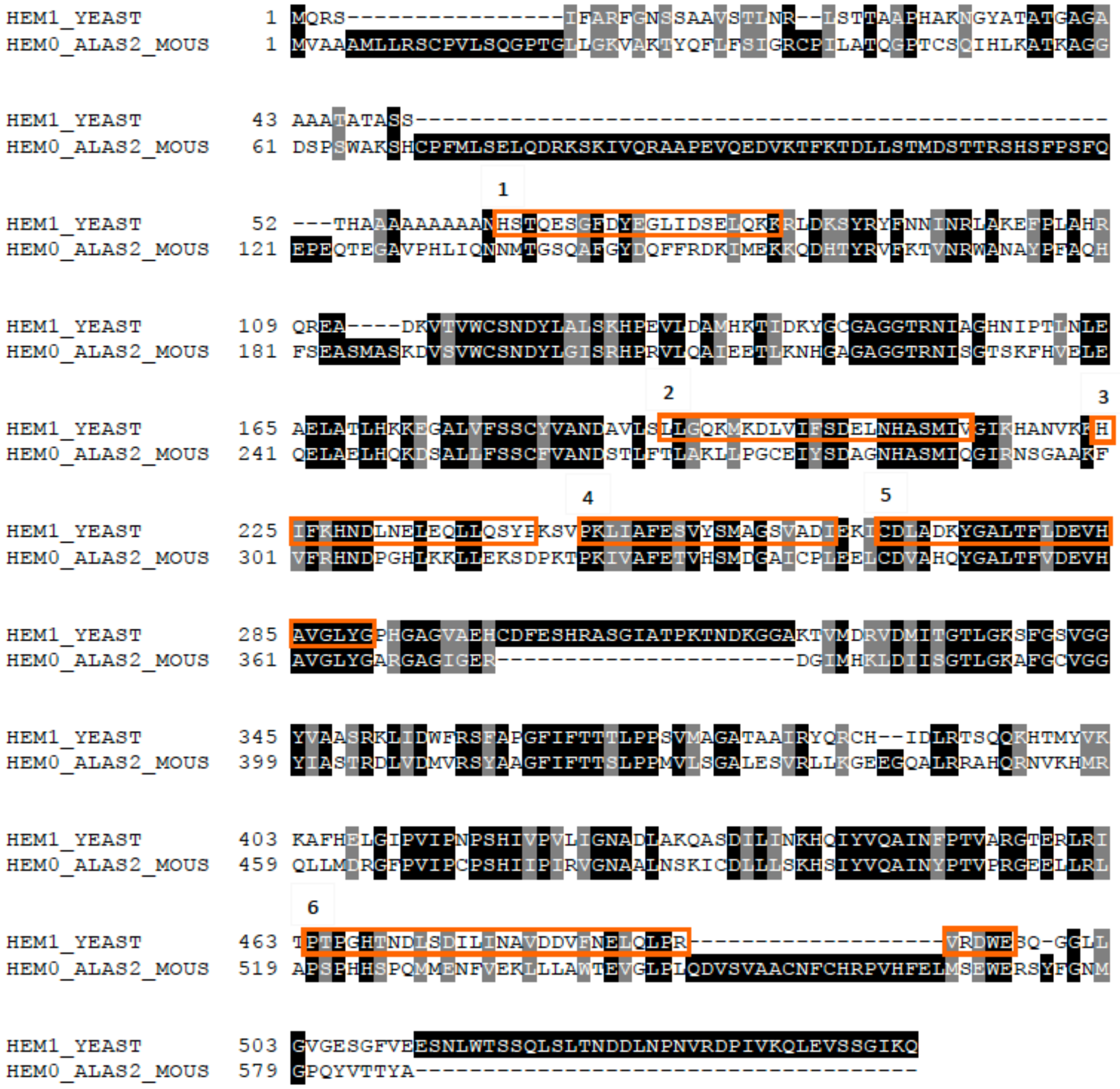

Figure S1. Alignment of *S. cerevisiae* Hem1 with mouse ALAS2. The boxed regions on yeast Hem1 are sequences that bound to Mcx1 (Clpx) (Kardon, Moroco, Engen, & Baker, 2020).

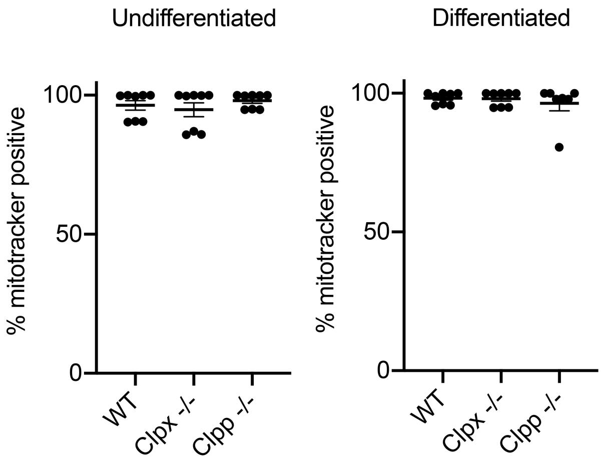

Figure S2. Mitotracker staining of WT, *Clpx -/-* and *Clpp -/-* MEL cells.

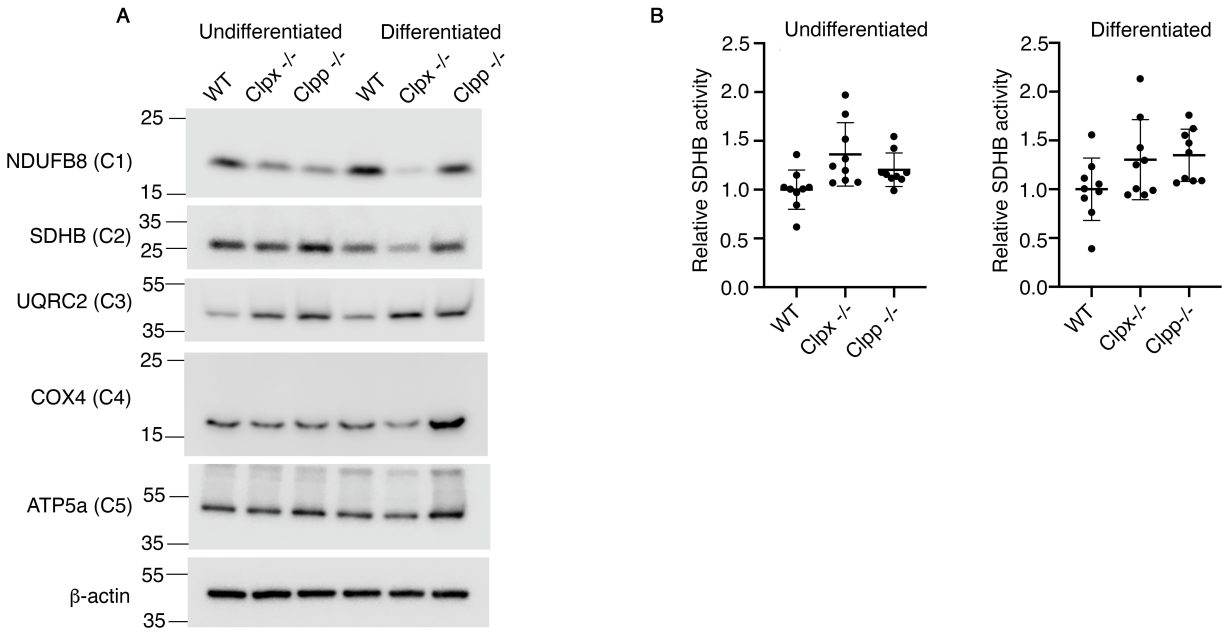

Figure S3. SDHB protein levels and activity are unaltered in *Clpx -/-* and *Clpp -/-* MEL cells. (A) Western blot of oxidative phosphorylation complex proteins. Fold change from Undifferentiated WT: NDUFB8: $Clpx^{-/-}$ = 0.534 ± 0.007 and $Clpp^{-/-}$ = 0. 589 ± 0.073, SDHB: $Clpx^{-/-}$ = 0.871 ± 0.047 and $Clpp^{-/-}$ = 0.953 ± 0.137, UQRC2: $Clpx^{-/-}$ = 1.924 ± 0.144 and $Clpp^{-/-}$ = 2.315 ± 0.417, COX4: $Clpx^{-/-}$ = 0.992 ± 0.290 and $Clpp^{-/-}$ = 0.901 ± 0.160, ATP5a: $Clpx^{-/-}$ = 1.017 ± 0.129 and $Clpp^{-/-}$ = 1.215 ± 0.057; from Differentiated WT: NDUFB8: $Clpx^{-/-}$ = 0.480 ± 0.209 and $Clpp^{-/-}$ = 0.918 ± 0.183, SDHB: $Clpx^{-/-}$ = 1.048 ± 0.273 and $Clpp^{-/-}$ = 1.254 ± 0.114, UQRC2: $Clpx^{-/-}$ = 2.618 ± 0.125 and $Clpp^{-/-}$ = 2.158 ± 0.136, COX4: $Clpx^{-/-}$ = 1.425 ± 0.625 and $Clpp^{-/-}$ = 1.661 ± 0.516, ATP5a: $Clpx^{-/-}$ = 1.264 ± 0.200 and $Clpp^{-/-}$ = 1.637 ± 0.212. N = 4. (B) SDHB activity assays in undifferentiated (left) and differentiated (right) MEL cells P values: Undifferentiated: WT vs Clpx-/- = 0.0008 and WT vs Clpp-/- = 0.0477; Differentiated: WT vs Clpx-/- = 0.0385 and WT vs Clpp-/- = 0.0171.

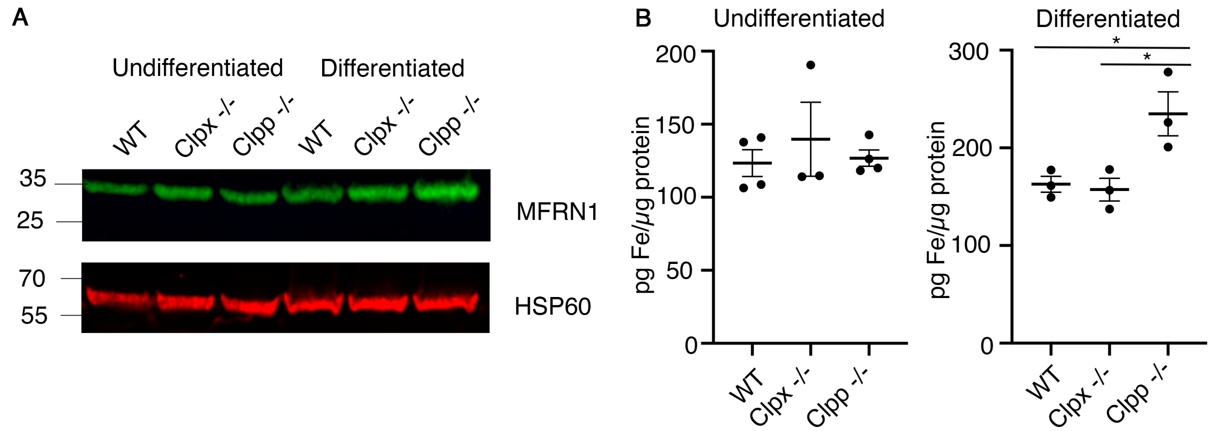

Figure S4. The iron defect in *Clpx*$^{-/-}$ cells is not caused by an iron transport defect. (A) Western blot analysis of MFRN1 levels in *Clpx*$^{-/-}$ and *Clpp*$^{-/-}$ MEL cells. Fold change from Undifferentiated WT: *Clpx*$^{-/-}$ = 1.141 ± 0.211 and *Clpp*$^{-/-}$ = 1.124 ± 0.265; from Differentiated WT: *Clpx*$^{-/-}$ = 1.083 ± 0.129 and *Clpp*$^{-/-}$ = 1.437 ± 0.290. N = 3. **(B)** ICP-MS analysis of iron levels in WT, *Clpx*$^{-/-}$ and *Clpp*$^{-/-}$ MEL cells. There is no significant decrease in iron levels in either *Clpx*$^{-/-}$ or *Clpp*$^{-/-}$ MEL cell mitochondria relative to WT. P values for differentiated WT vs. *Clpp*$^{-/-}$ iron levels: P= 0.0395; *Clpx*$^{-/-}$ vs. *Clpp*$^{-/-}$: P= 0.0377.

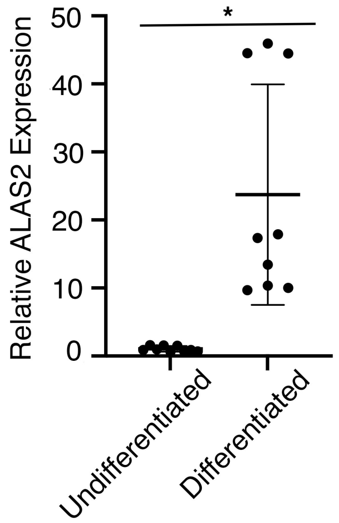

Figure S5. qPCR analysis of ALAS2 expression in undifferentiated vs. differentiated MEL cells. ALAS2 expression significantly increases during erythroid cell differentiation. P-value for differentiated vs undifferentiated: P=0.007.

Supplemental References

1. Grillo, A. S., SantaMaria, A. M., Kafina, M. D., Cioffi, A. G., Huston, N. C., Han, M., Seo, Y. A., Yien, Y. Y., Nardone, C., Menon, A. V., Fan, J., Svoboda, D. C., Anderson, J. B., Hong, J. D., Nicolau, B. G., Subedi, K., Gewirth, A. A., Wessling-Resnick, M., Kim, J., Paw, B. H., and Burke, M. D. (2017) Restored iron transport by a small molecule promotes absorption and hemoglobinization in animals. *Science* **356**, 608-616
2. Roy, C. N., Penny, D. M., Feder, J. N., and Enns, C. A. (1999) The hereditary hemochromatosis protein, HFE, specifically regulates transferrin-mediated iron uptake in HeLa cells. *J. Biol. Chem* **274**, 9022-9028
3. Yien, Y. Y., Robledo, R. F., Schultz, I. J., Takahashi-Makise, N., Gwynn, B., Bauer, D. E., Dass, A., Yi, G., Li, L., Hildick-Smith, G. J., Cooney, J. D., Pierce, E. L., Mohler, K., Dailey, T. A., Miyata, N., Kingsley, P. D., Garone, C., Hattangadi, S. M., Huang, H., Chen, W., Keenan, E. M., Shah, D. I., Schlaeger, T. M., DiMauro, S., Orkin, S. H., Cantor, A. B., Palis, J., Koehler, C. M., Lodish, H. F., Kaplan, J., Ward, D. M., Dailey, H. A., Phillips, J. D., Peters, L. L., and Paw, B. H. (2014) TMEM14C is required for erythroid mitochondrial heme metabolism. *J. Clin. Invest.* **124**, 4294-4304
4. Barman-Aksözen, J., Halloy, F., Iyer, P. S., Schümperli, D., Minder, A. E., Hall, J., Minder, E. I., and Schneider-Yin, X. (2019) Delta-aminolevulinic acid synthase 2 expression in combination with iron as modifiers of disease severity in erythropoietic protoporphyria. *Mol. Genet. Metab.* **128**, 304-308
5. Yien, Y. Y., Ducamp, S., van der Vorm, L. N., Kardon, J. R., Manceau, H., Kannengiesser, C., Bergonia, H. A., Kafina, M. D., Karim, Z., Gouya, L., Baker, T. A.,

Puy, H., Phillips, J. D., Nicolas, G., and Paw, B. H. (2017) Mutation in human CLPX elevates levels of delta-aminolevulinate synthase and protoporphyrin IX to promote erythropoietic protoporphyria. *Proc. Natl. Acad. Sci. U S A* **114**, E8045-E8052
6. Crooks, D. R., Ghosh, M. C., Haller, R. G., Tong, W. H., and Rouault, T. A. (2010) Posttranslational stability of the heme biosynthetic enzyme ferrochelatase is dependent on iron availability and intact iron-sulfur cluster assembly machinery. *Blood* **115**, 860-869
7. Maio, N., Kim, K. S., Holmes-Hampton, G., Singh, A., and Rouault, T. A. (2019) Dimeric ferrochelatase bridges ABCB7 and ABCB10 homodimers in an architecturally defined molecular complex required for heme biosynthesis. *Haematologica* **104**, 1756-1767
8. Fujimaki, M., Furuya, N., Saiki, S., Amo, T., Imamichi, Y., and Hattori, N. (2019) Iron Supply via NCOA4-Mediated Ferritin Degradation Maintains Mitochondrial Functions. *Mol. Cell. Biol.* **39**
9. Yien, Y. Y., and Bieker, J. J. (2012) Functional interactions between erythroid Krüppel-like factor (EKLF/KLF1) and protein phosphatase PPM1B/PP2Cβ. *J. Biol. Chem.* **287**, 15193-15204
10. Canver, M. C., Bauer, D. E., Dass, A., Yien, Y. Y., Chung, J., Masuda, T., Maeda, T., Paw, B. H., and Orkin, S. H. (2014) Characterization of genomic deletion efficiency mediated by clustered regularly interspaced short palindromic repeats (CRISPR)/Cas9 nuclease system in mammalian cells. *J. Biol. Chem.* **289**, 21312-21324
11. Cong, L., Ran, F. A., Cox, D., Lin, S., Barretto, R., Habib, N., Hsu, P. D., Wu, X., Jiang, W., Marraffini, L. A., and Zhang, F. (2013) Multiplex genome engineering using CRISPR/Cas systems. *Science* **339**, 819-823
12. Ran, F. A., Hsu, P. D., Wright, J., Agarwala, V., Scott, D. A., and Zhang, F. (2013) Genome engineering using the CRISPR-Cas9 system. *Nat Protoc* **8**, 2281-2308
13. Yien, Y. Y., Shi, J., Chen, C., Cheung, J. T. M., Grillo, A. S., Shrestha, R., Li, L., Zhang, X., Kafina, M. D., Kingsley, P. D., King, M. J., Ablain, J., Li, H., Zon, L. I., Palis, J., Burke, M. D., Bauer, D. E., Orkin, S. H., Koehler, C. M., Phillips, J. D., Kaplan, J., Ward, D. M., Lodish, H. F., and Paw, B. H. (2018) FAM210B is an erythropoietin target and regulates erythroid heme synthesis by controlling mitochondrial iron import and ferrochelatase activity. *J. Biol. Chem.* **293**, 19797-19811
14. Li, F., Lim, C. K., and Peters, T. J. (1987) An h.p.l.c. assay for protoporphyrinogen oxidase activity in rat liver. *Biochem. J.* **243**, 863-866
15. Bergonia, H. A., Franklin, M. R., Kushner, J. P., and Phillips, J. D. (2015) A method for determining δ-aminolevulinic acid synthase activity in homogenized cells and tissues. *Clin. Biochem.* **48**, 788-795
16. Peter, F., Growcock, G., and Strunc, G. (1978) Fluorometric determination of erythrocyte protoporphyrin in blood, a comparison between direct (hematofluorometric) and indirect (extraction) methods. *Clin. Chem.* **24**, 1515-1517
17. Rossi, E., Costin, K. A., and Garcia-Webb, P. (1988) Ferrochelatase activity in human lymphocytes, as quantified by a new high-performance liquid-chromatographic method. *Clin. Chem.* **34**, 2481-2485
